# Bibliometric and content analysis of *Journal of Educational Evaluation for Health Professions* in 2018

**DOI:** 10.3352/jeehp.2018.15.35

**Published:** 2018-12-28

**Authors:** Yera Hur

**Affiliations:** Institute of Medical Education, Hallym University College of Medicine, Chuncheon, Korea; Hallym University, Korea

Since 2004, *Journal of Educational Evaluation for Health Professions* (JEEHP) has made outstanding advances in its quality. From January 1 to December 27, 2018, a total of 100 manuscripts were submitted to JEEHP. There were 6 commissioned articles, including 4 editorials and 2 corrigenda. One article published in 2018 was submitted in 2017. Of the 94 unsolicited articles, 29 were published and 57 were returned without review as unsuitable or for other reasons, 4 were rejected after review, 2 were withdrawn, and 2 remained under review. The acceptance rate in 2018 was 31.5%, which is similar to the rate of 27.4% from 2017 [1].

Of the 36 articles published in 2018, there were 15 research articles, 5 opinions, 4 case reports, 4 brief reports, 1 review, 1 software report, 4 editorial, and 2 corrigenda.

Medicine (18 articles) was the most common discipline, followed by dentistry (4 articles), health care in general (4 articles), and physical therapy (3 articles). This pattern is quite different from that observed in 2017, when more articles were published from diverse disciplines, such as occupational therapy and pharmacy (Fig. 1).

In the majority of the studies, the subjects were students (n= 20), followed by residents (n= 2), faculty members (n= 1), and a program itself (n= 1) (Supplement 1). The rest of the articles could not be categorized by subject. The tendency for most articles to utilize students as the main subjects continues from last year’s publications, which also focused on education from a student-centered perspective.

The research content of the articles also showed some differences from last years’ papers. As shown in Fig. 2, the articles published this year were mainly about course evaluations (n= 12), followed by perception surveys (n= 7), computer-based test/computerized adaptive testing/licensing examinations (n= 6), and assessment tools (n= 3). In contrast, most papers from 2017 dealt with perception surveys, whereas in 2018 there was a remarkable increase in the number of articles on licensing examinations and computerized adaptive testing (Fig. 2).

Of the 30 unsolicited articles that were published, only 1 was a qualitative study. This trend did not change from last year, but it does not mean that qualitative methods of evaluation should be ignored. I hope that more researchers use qualitative methods in their research in the future.

One of the features that shows a journal’s diversity is the variety of countries of authors. Last year, the first authors were from 11 countries. This year, the first authors were from 9 different countries: the authors of 14 articles were from Korea, followed by the United States (n= 10) (Fig. 3). This trend did not change from last year; however, the distribution of other nationalities was different. Only 1 article was written by authors from 2 countries [2].

There were 4 major features of the articles published in 2018 compared to those published in 2017. First, the majority of articles dealt with medical students. This trend did not change from last year. Second, the research content of this year was mainly focused on course evaluations. There was a remarkable increase in articles on licensing examinations and computerized adaptive testing/computer-based testing. Third, like last year, authors from Korea and United States accounted for most of the publications; however, papers were also published by authors from a variety of countries. Lastly, many researchers preferred to work collaboratively, but most authors worked with colleagues from the same country, except for 1 article with authors from Chile and the United Kingdom [2].

Regardless of the number of manuscripts submitted, I hope that the journal will maintain its low acceptance rate in order to preserve its high quality; publish more diverse content with diverse methodologies, including qualitative studies; and receive the manuscripts of multi-national studies.

## Figures and Tables

**Fig. 1.**
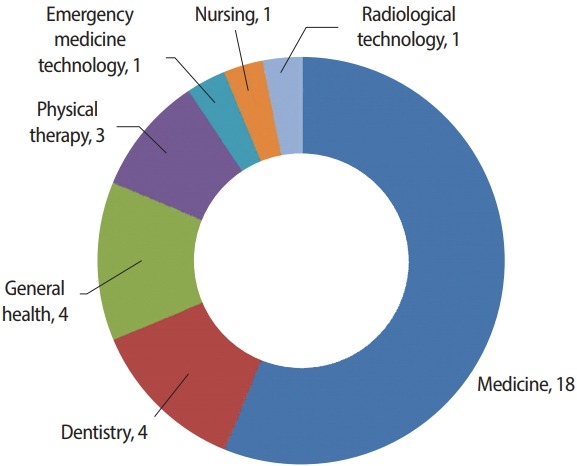
Classification of the 31 articles published in *Journal of Educational Evaluation for Health Professions* in 2018 according to discipline.

**Fig. 2.**
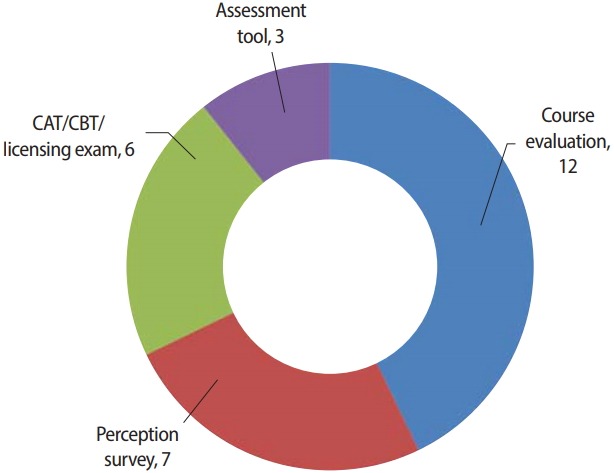
Number of articles published in *Journal of Educational Evaluation for Health Professions* in 2018 by research content. CAT, computerized adaptive test; CBT, computer-based testing.

**Fig. 3.**
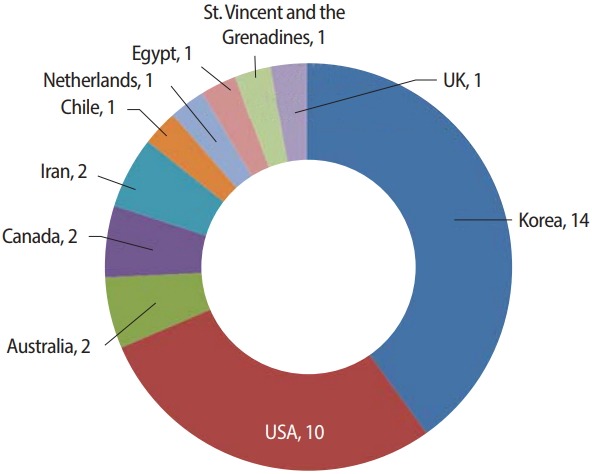
Country of authors’ affiliation of 33 articles (excluding 2 corrigenda) published in *Journal of Educational Evaluation for Health Professions* in 2018.

## Supplementary material

Supplement 1. Raw data file of the analysis of the articles in the 2018 issues of *Journal of Educational Evaluation for Health Professions*.
